# Transpseudarthrosis Osteotomy with Interbody Fusion for Kyphotic Spinal Pseudarthrosis in Ankylosing Spondylitis by a Single Posterior Approach: A Retrospective Study and a Brief Relevant Literature Review

**DOI:** 10.1155/2017/4079849

**Published:** 2017-08-10

**Authors:** Erzhu Yang, Liangliang Cao, Guowang Zhang, Xiaofeng Lian, Jianguang Xu

**Affiliations:** Department of Orthopedics, Shanghai Jiaotong University Affiliated Sixth People's Hospital, No. 600 Yishan Rd, Shanghai 200233, China

## Abstract

**Objective:**

To explore the safety and efficacy of transpseudarthrosis osteotomy with interbody fusion in the treatment of Ankylosing Spondylitis (AS) patients with kyphotic spinal pseudarthrosis by a single posterior approach.

**Methods:**

Twelve consecutive patients with spinal pseudarthrosis underwent transpseudarthrosis osteotomy and interbody fusion with a polyetheretherketone (PEEK) cage by a single posterior approach. The operative time, intraoperative blood loss, and complications were recorded. Radiographic and clinical results were assessed preoperatively and at the final follow-up.

**Results:**

The average operative time was 201.9 min and the mean blood loss was 817.5 ml. The visual analogue scale (VAS) improved significantly from 6.7 preoperatively to 1.1 at the final follow-up. The average correction of the segmental kyphosis at the level of the pseudarthrosis was 22.3°. Bony fusion was achieved in all patients, and there was no obvious loss of correction at follow-up.

**Conclusion:**

Transpseudarthrosis osteotomy at the level of the pseudarthrosis can be safely performed and surgical repair of pseudarthrosis with interbody fusion by a single posterior approach was feasible.

## 1. Introduction

Chronic inflammatory rheumatic disease affects the axial skeleton, causing characteristic inflammatory back pain, which leads to structural and functional impairments and a decrease in the quality of life [1]. Similar to many other chronic inflammatory rheumatic diseases, AS targets bones [2]. The interaction between inflammation and bones is characterized by a wide range of changes in bone remodeling, including not only new bone formation in the form of syndesmophytes but also erosions, generalized osteoporosis, and vertebral fractures [3, 4]. Fractures of the ankylosed and osteoporotic vertebral column in patients with AS are relatively common after trivial trauma [5–8]. The initial injury is often overlooked because of the history of chronic pain in the back before the trauma. Without proper immobilization and persistent abnormal motion, the overlooked injury site may develop extensive discovertebral destruction and kyphotic deformity, which may cause localized pain and neurological abnormalities [9–11]. This kind of destructive discovertebral lesions or pseudarthrosis, with radiographically evident sclerosis and destruction around the disc margins, is also known as Andersson lesion (AL) and was first described by Andersson [12] in 1937. Surgical intervention is widely accepted in patients with AL, who present with painful thoracolumbar kyphosis, neurological deficits, and sagittal imbalance [13–17]. Several previous series have reported the clinical and radiographic outcome of different wedge osteotomies with supplemental anterior fusion in patients with kyphotic AL-complicated AS [13, 15–18]. However, there is controversy regarding the optimal surgical procedure. To the best of our knowledge, few studies have focused on the feasibility and outcome of transpseudarthrosis osteotomy with interbody fusion for treatment of kyphotic AL-complicated AS by a single posterior approach. The purpose of this study is to evaluate the effectiveness and safety of this technique for correcting and stabilizing kyphotic AL-complicated AS.

## 2. Material and Methods

### 2.1. Patients

From 2009 to 2013, twelve consecutive patients with AS and pseudarthrosis at the thoracolumbar segment, as confirmed with established clinical and radiographic criteria, were treated in our department. The group consisted of 10 male and 2 female patients with a mean age of 52.2 years (range 38–61 years). At the time of diagnosis of the pseudarthrosis, all 12 patients complained of localized back pain. Nine patients related their acute pain to a fall to the ground with direct impact on the back or motor bicycle accident. One patient presented with localized pain around the pseudarthrotic lesion after a severe cough. The other two patients denied having suffered any trauma. Of these patients 5 had a neurologic deficit of Frankel grade D and 1 patient of Frankel grade C (Table 1).

### 2.2. Laboratory Evaluation

Before surgery, the possible associations between systemic inflammatory response and AS, laboratory studies, including erythrocyte sedimentation rate (ESR), C-reactive protein (CRP), and human leukocyte antigen B27 (HLA-B27), were evaluated.

### 2.3. Radiologic Assessment

**Figure 1:**
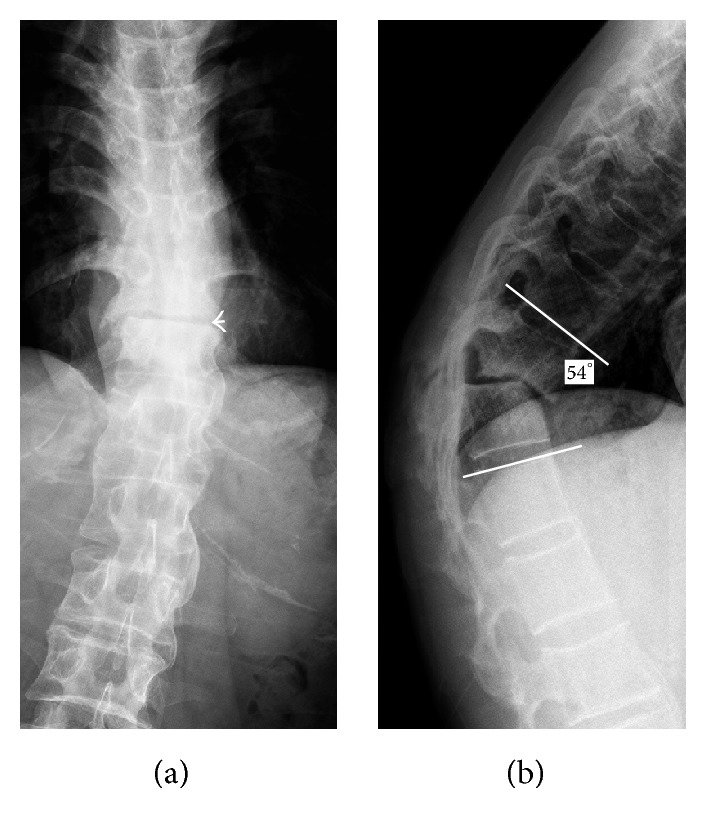
AP and lateral radiographs of spinal pseudarthrosis in AS. Radiographs showed a rigid spine with pseudarthrosis (a, arrow) at the T11-T12 level and a local kyphosis of 54° (b).

**Figure 2:**
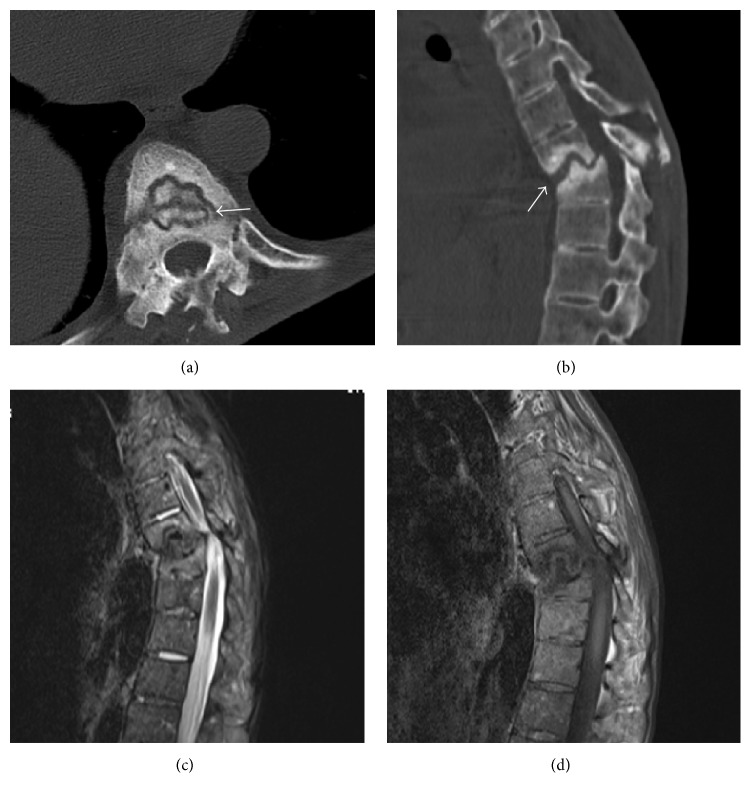
A 54-year-old female patient presented with painful round kyphosis and motor loss of bilateral lower extremities. CT scan showed destruction of the vertebral body and vacuum phenomenon (a, arrow). CT sagittal reconstruction image (b) demonstrated irregular bony gap (arrow) from anterior to posterior column and sclerosis of adjacent vertebral bodies. T2- (c) and T1-weighted (d) sagittal images revealed low signal at the pseudarthrosis and compression of the spinal cord.

All patients were referred for X-rays, CT, and MRI examinations. AP and lateral radiographs demonstrated a rigid spine with a thoracolumbar kyphotic angulation (Figure 1). CT scans demonstrated irregular discovertebral osteolysis with reactive sclerosis and an extraordinary large vacuum phenomenon (Figure 2). MRI showed bony exophytic element with narrowing of the canal at this level of pseudarthrosis (Figure 2). Standing AP and lateral radiographs were obtained before and after surgery and at last follow-up.

### 2.4. Surgical Procedure

All patients were operated on under the general anesthesia, and all procedures were performed while monitoring somatosensory-evoked potentials. Briefly, a standard midline exposure was performed. Pedicle screws were then inserted in at least three levels above and three levels below the lesion. Initially, the irregular osteophytes along with the inferior aspect of the spinous process above and the superior aspect of the spinous process below were excised. The bilateral inferior facets of the cephalad level and superior facet of the caudad level were resected flush with pedicles above and below the pseudarthrosis. The lower half of the lamina of the cranial vertebra was removed to expose the dura and nerve roots. Then, the canal was enlarged centrally and a portion of the floor and the roof of the intervertebral foramen were removed to accommodate the dura and the roots during the correction, especially for patients with spinal cord compression at the apex of the kyphosis. During the partial laminectomy and the enlargement of the central canal it was important to separate the dura carefully because it was always adherent to the degenerated ligamentum flavum or directly to the lamina.

**Figure 3:**
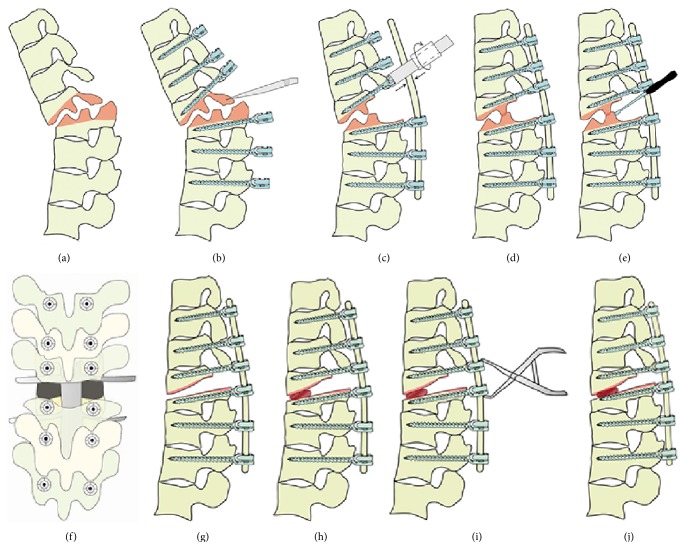
The procedure of transpseudarthrosis osteotomy with interbody fusion. (a) The pseudarthrosis lesion area of an ankylosed spine. (b) Posterior elements with irregular osteophytes along with bilateral inferior and superior facet were resected flush with pedicles above and below the pseudarthrosis and the canal was enlarged. (c) A temporary rod was inserted into the screws to maintain the stability. (d–g) The transpseudarthrosis osteotomy was performed using an osteotome, rongeurs, curettes, or high-speed drill. (h) A PEEK cage with autograft inside was carefully inserted into the intervertebral space. (i-j) The final correction was achieved by slowly extending the reduction operating table in combination with compressive pressure on the pedicle screws above and below the inserted cage.

The pseudarthrotic cavity with fibrous tissue, fibrocartilage, and necrotic bone was removed using an osteotome, rongeurs, curettes or high-speed drill. With the posterior border of the line between the base of pedicles of the same vertebra and the midpoint of the anterior margin of the pseudarthrosis cavity as the apex of the osteotomy, an oblique osteotomy was performed at both the cranial and caudal vertebrae. This osteotomy was performed from the lateral direction under direct vision of the spinal cord. Then, bilateral distraction through the intervertebral gap was performed and a PEEK cage with autograft inside was carefully inserted into the intervertebral space (Figure 3).

### 2.5. Outcome Assessment

Clinical outcomes were assessed by visual analogue scale (VAS) for back pain and Frankel neurological classification for neurological status preoperatively and at the final follow-up.

### 2.6. Statistical Analysis

Data were expressed as mean ± standard deviations for variables. Preoperative and postoperative differences were performed using paired *t*-test, and a *P* value of *P* < 0.05 was taken to indicate statistical significance. All data analysis was done using Statistical Package for Social Sciences (SPSS), Version 17.0 for windows.

## 3. Results

### 3.1. Laboratory Results

The HLA-B27 test was positive in all patients. Only one patient showed normal ESR, and the other six patients were above the normal range. All patients had raised CRP levels (Table 1). Histopathologic examination of the excised specimen revealed degenerated fibrocartilage with diffuse fibrosis and nonspecific chronic inflammatory cell infiltration.

### 3.2. Radiologic Results

**Figure 4:**
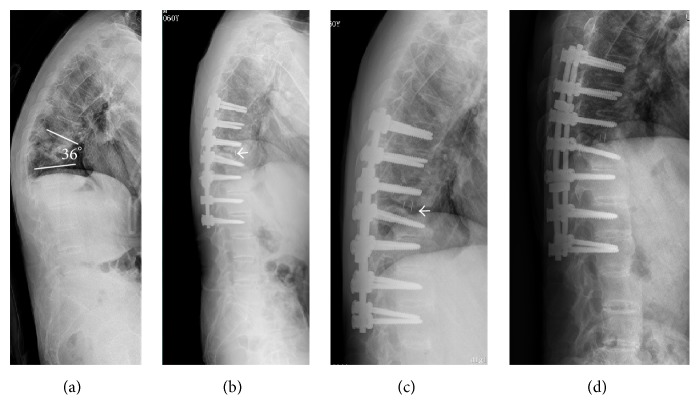
A 59-year-old male patient presented with severe back pain and progressive kyphotic deformity. Lateral radiograph (a) before surgery showed local kyphosis of 36° and pseudarthrosis at T10-T11 level. Immediately after surgery lateral X-ray (b) revealed transpseudarthrosis osteotomy and interbody fusion with PEEK cage (arrow). Three months after surgery, normal sagittal alignment was maintained and partial union (c, arrow) was obtained. At final follow-up, no evidence of nonunion and no loss of correction on lateral X-ray (d).

The average follow-up was 4.9 years (range, 3–7 years). The local kyphosis was corrected from 30.3° (range, 9°–53°) preoperatively to 8.0° (range, 1°–21°) postoperatively, with a mean correction of 22.3° (Table 2). The mean time for radiologic union of pseudarthrosis was 4.2 months (range, 3–6 months) and none of the patients had any loss of correction or recurrent pseudarthrosis at the final follow-up (Figure 4).

### 3.3. Surgical and Clinical Results

The average operative time was 201.9 min (range, 170–260 min). The mean blood loss was 817.5 ml (range, 530–1460 ml). All patients showed significant improvement of back pain after surgery. The VAS improved significantly from 6.7 preoperatively to 1.1 at the final follow-up (Table 2). Five patients with a neurological deficit (Frankel D) preoperatively had improved to Frankel E at the last follow-up. Only one patient (Patient 3, Table 2) with a neurological deficit (Frankel C) preoperatively still had motor deficiencies (4/5) in both legs at the final follow-up, but he was satisfied with the surgical results for the improvement of back pain.

### 3.4. Complications

There was one intraoperative dural tear, which was repaired primarily and did not result in sequelae. Screws pullout happened in 1 patient and they were enhanced with bone cement augmentation. There were no neurovascular complications after posterior surgery in our series. No infections occurred and no evidence of screw failures or nonunions at the level of pseudarthrosis was observed at the final follow-up.

## 4. Discussion

Spinal pseudarthrosis in patients with AS is extremely unstable because the entire vertebral column is a brittle, osteoporotic unit and the calcified supporting ligaments are also broken, somewhat like a long bone fracture [19]. These destructive lesions may cause sagittal plane imbalance leading to progressive kyphotic deformity and may be complicated by severe back pain and neurological deficits [15–18]. In the current series, all patients presented with localized pain, and six of them developed thoracolumbar kyphosis, with five of them having neurological deficits. Surgical intervention is widely accepted in patients with AL, who present with painful thoracolumbar kyphosis, neurological deficits, and sagittal imbalance.

Chang et al. [17] reported that pseudarthrosis can be treated effectively by posterior correction and fixation at the level of pseudarthrosis without anterior fusion. Chang et al. [17] presented 30 patients that were treated with OWOs at the level of the pseudarthrosis without anterior fusion and achieved a mean of 38° correction for the local kyphosis. In addition in the series by Chang et al. [17] none of the patients had any notable loss of correction, and fusion was observed at the anterior gaps created by OWO at the final follow-up. Chang et al. [17] had promising results regarding the superior fusion ability in patients with AS. However, Cho et al. [20] reported that the mean correction of the kyphotic angle at the osteotomy sites for the OWOs was 24.9°, corresponding to 10.7° per segment and, for those with three or more OWOs, the mean correction was 33.0°. Kim et al. [15] reported that the average correction of segmental kyphosis was 20.9° at the level of pseudarthrosis with OWO. Theoretically, this procedure [17] would create a big anterior gap, and without anterior fusion the posterior implants would be under considerable tension, thereby increasing the risk of implant failure, delayed union or nonunion, and inevitable loss of correction. In a previous research, 3 rods broke at the osteotomy site because of delayed union after OWO [21]. Chang et al. [17] observed an average 2° loss of correction at the local kyphosis during the follow-up period.

The sharp lordotic angle and elongation of the anterior column occurring in OWO were associated with serious vascular and neurological complications. To avoid such complications CWOs were introduced. Thomasen [22] first described this technique, which was described as a V-shaped wedge resection of the vertebral body, including both pedicles and posterior elements. Correction was achieved by passive extension of the spine, thus closing the posterior osteotomy with an anterior hinge. As this procedure did not lengthen the anterior column, there should not be lengthening of structures anterior to the spine. In fact, the spine would be shortened. The technique did not create an anterior bony defect, potentially reducing the need for an anterior procedure and providing more stable correction.

Qian et al. [18] reported a series of 7 AS patients with thoracolumbar pseudarthrosis and kyphotic deformity that underwent PSO at the level of pseudarthrosis. A second-stage supplemental anterior fusion was performed two weeks after the posterior procedure. In the study by Qian et al. [18], the correction of global kyphosis and local kyphosis following PSO was 45° and 36.2°, without neurological complications. All patients achieved solid fusion without any loss of correction at the final follow-up. This satisfactory radiographic and clinical outcome was complicated with a mean blood loss of 2,200 ml during the PSO procedure and 470 ml during the second-stage supplemental anterior fusion. We also noticed that, in the series by Qian et al., after PSO, the pseudarthrotic cavity was still a bony defect in the anterior and middle column even if the technique itself did not create an anterior bony defect. This was one of the several reasons for a second-stage supplemental anterior fusion in the article by Qian et al. [18]. PSO could achieve great correction at a single segment, with an average correction of 30° to 40°. In addition, this procedure resulted in a greater amount of blood loss [16, 18, 20, 21, 23, 24].

Kim et al. [15] performed a series of SPO only at the level of pseudarthrosis for 4 cases, and the other 8 patients were treated with SPO and PSO. All cases were treated with anterior interbody fusion in one-stage or two-stage procedures. The mean correction was 20.9 with SPO and 26.3 with PSO. We noticed that deformity correction at the pseudarthrosis segments with SPO was only limited to a small degree, and they [15] performed additional PSO at the segment below L1 for cases with severe kyphosis.

A recent study [13] reported that they perform anterior fusion and posterior internal fixation for six AS patients with AL and the clinical outcome was satisfactory.

In those previous surgical series [13, 15, 18], authors paid more attentions to the ability of correction and the need for anterior fusion. However, we are more concerned about whether a patient with long-standing AS could tolerate so many complex surgical procedures, especially a combined anterior and posterior approach.

Compared with these studies [13, 15, 17, 18] (Table 3), we performed transpseudarthrosis osteotomy with interbody fusion through a single posterior approach. All patients showed significant improvement of back pain after surgery. The local kyphosis was corrected from 30.3° (range, 9°–53°) preoperatively to 8° (range, 1°–21°) postoperatively, with a mean correction of 22.3°. In contrast to PSO, which include resection of pedicles and the osteotomy through the osteoporotic vertebral body, the transpseudarthrosis osteotomy, which refers to removing fibrous tissue, fibrocartilage, and necrotic bone around the pseudarthrosis cavity, could significantly decrease the blood loss. In addition, the soft tissue including the epidural vein under the chronic compression of the apex of the kyphosis degenerated and calcified and the bone removed near the line between the pedicles was also sclerotic, which reduced epidural hemorrhage.

In our series, the mean blood loss was 817.5 ml (range, 530–1460 ml). The blood loss in our procedure was significantly lower than in the PSO procedure in the series by Qian et al. [18] and in the literature [20, 21, 24]. We also performed interbody fusion after osteotomy and distraction through the intervertebral gap. The spinal cord was identified under direct vision from the lateral side while the PEEK cage was carefully inserted into the prepared intervertebral space. This interbody fusion for pseudarthrosis allowed for direct repair of the lesion, and even before union the PEEK cage could offer enough support for the anterior column. Therefore, patients could obtain immediate stability after surgery, and there was no need to perform anterior surgery around the thoracolumbar junction.

Several surgeons [15, 16] indicated that the goals of surgical treatment of pseudarthrosis in AS patients are correction of the kyphotic deformity and maintenance of the corrected position. As numerous spinal osteotomies have been developed and combined with different approaches, these goals could be achieved. However, old patients with long-standing AS cannot always tolerate these complicated approaches, especially a combined anterior and posterior approach. Similar to Zhang et al.'s [25] report, by one-stage transpseudarthrosis osteotomy, correction of kyphosis deformity and fusion of the lesion plane were achieved with less blood loss and less operating time, which lead to less operative risk.

The purpose of this study is not to show the correction ability of transpseudarthrosis osteotomy for local kyphosis. This study tries to show that satisfactory radiological and clinical outcomes could be obtained by a single posterior approach for treatment of kyphotic AL-complicated AS.

## 5. Conclusion

We propose an alternative surgical option for treatment of kyphotic AL-complicated AS. This single posterior procedure is a safe and effective technique for correcting the sagittal imbalance and preventing an additional anterior approach. Surgical repair of pseudarthrosis with interbody fusion by a single posterior approach is feasible and results in sound union.

## Figures and Tables

**Table 1:** Summary of AS patients date before operation.

Patients number	Age (y)/sex	Level of lesion	History of injury	HLA-B27^*∗*^ (+/−)	ESR^†^ (mm/h)	CRP (mg/L)
1	38/M	T12-L1	MBA	+	28	14
2	52/M	T11-T12	Fall	+	35	23
3	47/M	T11-T12	Fall	+	30	15
4	60/M	T12-L1	Coughing	+	32	22
5	58/M	T12-L1	Fall	+	29	20
6	41/M	T11-T12	Fall	+	32	19
7	61/M	T12-L1	Fall	+	19	12
8	54/F	T11-T12	Fall	+	27	16
9	59/M	T10-T11	None	+	33	17
10	48/M	T12-L1	Fall	+	20	11
11	58/F	T11-T12	Fall	+	26	13
12	50/M	T12-L1	None	+	31	16

^*∗*^Normal value ranges from 0 to 21 mm/h. ^†^Normal value ranges from 0 to 10 mg/L; MBA indicates Motor Bicycle Accident.

**Table 2:** Clinical data of AS patients before operation and after operation.

Patients number	Operation time (min)	Blood loss (ml)	Pain score (VAS) pre-op/post-op	Neurological status (Frankel grade) pre-op/post-op	Local kyphosis (degree) pre-op/post-op	Complications
1	260	1460	8/1	D/E	38/7	None
2	210	720	6/0	E/E	25/9	None
3	180	820	7/2	C/D	41/21	Dura tears
4	190	790	6/1	D/E	38/12	None
5	170	580	5/1	E/E	9/1	None
6	195	980	7/1	D/E	12/4	None
7	240	1100	7/1	E/E	53/15	Screws pullout
8	178	600	6/0	D/E	34/2	None
9	182	850	8/3	E/E	21/8	None
10	200	730	7/1	D/E	40/8	None
11	198	530	6/1	E/E	17/1	None
12	220	650	7/1	E/E	36/8	None
Mean ± SD	201.9 ± 26.79	817.5 ± 261.5	6.67 ± 0.89/1.08 ± 0.79		30.33 ± 13.38/8.0 ± 5.92	
*P*			<0.001^*∗*^		<0.001^*∗*^	

VAS indicates visual analogue scale. ^*∗*^Compared with preoperative value using paired *t*-test.

**Table 3:** Results of different osteotomy for spinal pseudarthrosis complicating AS reported in literature and in the current study.

Author (year)	Surgical method	Clinical/radiological outcome	Blood Loss	Complications	Approach
Zhang and Zheng [13]	OWO without anterior fusion	A mean correction of 38° for local kyphosis	Not mentioned	Postoperation pneumonia in 1 patient	Posterior approach
Kim et al. [15]	SPO or PSO with anterior interbody fusion in one stage or two stages	The mean correction was 20.9° with SPO and 26.3° with PSO	Not mentioned	Dura tears in 3 patients	Combined posterior and anterior approach
Chang et al. [17]	PSO through pseudarthrosis with two-stage anterior interbody fusion in two stages	A mean correction of 36.2° for local kyphosis	A mean blood loss in PSO was 2200 ml and 470 ml in anterior fusion	None	Combined posterior and anterior approach
Qian et al. [18]	Anterior fusion and posterior internal fixation	A mean correction of 12° for local kyphosis in intervertebral space fracture patients	Not mentioned	None	Combined posterior and anterior approach
Current study	posterior reduction and transpseudarthrosis osteotomy with interbody fusion	The mean correction of 22.3° for local kyphosis	The mean blood loss was 817.5 ml	Dura tears in 1 patient and screws pullout in 1 patient	Posterior approach
